# Barriers and facilitators of incorporating Ubuntu principles in the integrated management of childhood illness

**DOI:** 10.4102/phcfm.v17i1.4802

**Published:** 2025-06-03

**Authors:** Felicia O. Meno, Fhumulani M. Mulaudzi, Nombulelo V. Sepeng

**Affiliations:** 1 Department of Nursing, Faculty of Health Sciences, University of Pretoria, Pretoria, South Africa

**Keywords:** barriers, caregivers, facilitators, IMCI, Ubuntu

## Abstract

**Background:**

Success in incorporating Ubuntu principles in the integrated management of childhood illness (IMCI) requires collaboration between health professionals and families and assistance from their communities. Despite this, the literature reviewed is silent about exploring caregivers’ perspectives regarding the barriers and facilitators of incorporating Ubuntu principles in managing childhood illness.

**Aim:**

The study explored and described the barriers and facilitators of incorporating Ubuntu principles in managing childhood illness.

**Setting:**

The study was conducted in selected primary healthcare settings, community health centres and clinics in the Mafikeng sub-district of the North West province.

**Methods:**

The study was conducted using exploratory descriptive contextual, qualitative design. The 36 participants were selected using purposeful sampling. Data were collected through focus group discussions, and thematic analysis was used to analyse the data.

**Results:**

The study revealed three themes: the negative attitude of professional nurses, communication barriers and facilitators enhancing the incorporation of Ubuntu into IMCI. The findings indicated that health education of caregivers is crucial, the right allocation of nurses will facilitate the inclusion of Ubuntu into IMCI and the unannounced visit of government authorities will also facilitate the incorporation.

**Conclusion:**

The study illustrated that caregivers face several barriers hindering the incorporation of Ubuntu in IMCI. These findings support the need for healthcare providers to prioritise the incorporation of Ubuntu principles for better management of childhood illness for children under the age of five.

**Contribution:**

This is the first study to report the barriers and facilitators of incorporating Ubuntu principles into IMCI.

## Introduction

Globally, efforts are needed to reduce childhood mortality and foster children’s optimal growth and development rather than solely emphasising access to healthcare. To achieve this, the World Health Organization (WHO), the United Nations Children’s Fund (UNICEF) and other partners developed the Integrated Management of Childhood Illness (IMCI) in the mid-1990s.^1^ In 1998, South Africa became one of the 100 nations that adopted and implemented IMCI as a strategy in primary healthcare facilities.^2^ However, the success of IMCI in providing quality care has been achieved because of the involvement of caregivers. Meno, Makhado and Matsipane report that the success of IMCI necessitates collaboration between health professionals and families with assistance from their communities.^3^ They further state that families are primarily obligated to provide care and show the most significant concern for their children.^3^ This indicates that Ubuntu principles should be incorporated into IMCI to enhance the quality of consulting for children under 5 years old in primary healthcare facilities. Ubuntu is a dominant ethical thinking in African intellectual tradition, meaning *umuntu umuntu ngabantu*, which means ‘a human being is through their relationship with others’. This best expresses the emphasis on communal relationships.^4^ This philosophy emanates from the word *ubuntu,* which means being human, in Southern Nguni languages, and it is common among, including amaZulu, amaXhosa, MaSwati and amaNdebele. Bapedi, Basotho and Batswana have a similar phrase ‘*botho*’.

Ubuntu philosophy has different principles.^5^ Mugumbate and Chereni explain that African people believe Ubuntu as a set of principles and practices is essential for becoming real humans.^6^ Although attitudes and customs differ among ethnic groups, they all share a common theme. Ngubane and Makua highlight that compassion is likely one of the most significant principles of the Ubuntu philosophy and indigenous African way of life.^5^ Ubuntu indicates collectivism, solidarity, collaboration, participatory decision-making and a sense of belonging when utilised in healthcare.^7^

Ubuntu principles have been incorporated into different interventions to promote quality care among Africans.^4^ This indicates that the absence of relationship ties can reduce good health. Ubuntu is marked by a system of mutual trust and respect, and is considered an important feature of a relationship created over time and anchored in solidarity, reciprocity and accountability.^8^ The confidence in another person’ behaviour is the trust that Ubuntu fosters via long-term, open, kind and respectful interpersonal interactions. For instance, community members would believe medical advice from professional nurses.^9^ Working together is another fundamental principle of Ubuntu’s philosophy. It fosters togetherness and team spirit among individuals as they solve issues. Ubuntu promotes unity and oneness among group members.^5^ Incorporating Ubuntu can help address inequity, as there will be a sense of belonging and support for each other.^7^

Contrary to these, studies have revealed somewhat toxic relations between nurses and patients. The current study was conducted at a time when there were reports about nurses neglecting patients’ needs and dehumanising patients, especially in primary healthcare settings in public healthcare facilities.^10^ Whereby nurses failed to uphold Ubuntu principles when caring for under-5-year-old children.^11^ This indicates a lack of Ubuntu, compassion for others, and caring, sharing, warmth and understanding. In healthcare settings, nurses facilitate the incorporation of Ubuntu into IMCI to improve patient care and encourage holistic care for children under 5 years old.^11^ However, there could be barriers hampering the incorporation of Ubuntu into IMCI. Studies have reported caregivers’ complaining about nurses when taking their children for IMCI. The study aimed to explore and describe the caregivers’ perspectives on the barriers and the facilitators of incorporating Ubuntu principles into IMCI for children under the age of five.

## Research methods and design

### Study design

A qualitative exploratory descriptive contextual design was followed to explore and describe the caregiver’s perspectives on the barriers and facilitators of incorporating Ubuntu principles in management of childhood illness. In order to further grasp the nature of the problem, an exploratory research methodology was employed.^12^ The descriptive research aims to shed light on current issues through data collection process that allows caregivers to be fully descriptive and go into detail about the phenomena. Furthermore, the descriptive research design was used to capture the most authentic and accurate description of data collected to describe the caregiver’s perspective on the barriers and facilitators of incorporating Ubuntu principles into IMCI.^13^ The environment where data were collected was free from manipulation with natural surroundings.

### Study setting

The study was conducted in public primary healthcare (PHC) facilities, which are two community health care (CHC) centres and three clinics in the Mafikeng sub-district region of the North West province. The researchers choose these facilities because they have high headcounts on children under the age of five. The two CHC centres operate for 24 h, and the three clinics operate for 8 h a day. The study was conducted in the setting preferred by caregivers because, during the planning of the data collection, they indicated that it would be advantageous for the researcher to collect data where those under 5 years old are cared for at their convenience as those clinics and CHC centres are near their homes.

### Study population and sampling strategy

The target population of the study consisted of caregivers of children under 5 years old seeking care in PHC facilities. The inclusion criteria included any caregivers and/or guardians caring for a child under 5 years old and consulting in nearby clinics or CHC centres in Mafikeng sub-districts. Non-probability purposive sampling was used to sample eligible caregivers who would participate in the study. The caregivers who had just given birth and nursing children for less than a month were excluded from participating in the study. The sample size was achieved through data saturation after conducting six focus group discussions (FGDs) with 36 caregivers in different facilities.

### Data collection

The researcher used FGDs with more than eight caregivers in each facility to collect data. Identification and engagement of caregivers as a first step was achieved. The caregivers were 18 years and older, and brought their children for immunisation services at the identified PHC facilities in the Mafikeng sub-district. The purpose of the study was explained to the caregivers in their language of preference, which mainly was Setswana. The researcher then explained to the caregivers the questions that would be asked. A research question emerged from the caregivers answers based on their concerns even though the researcher had a research question in mind. The informed consent was obtained from caregivers, and the information sheet was written in Setswana. This was to ensure that caregivers understand their rights, roles and the implications of their participation in the study, allowing them to make more informed decisions. The central question posed was: what are caregivers’ views on barriers to incorporating Ubuntu principles in IMCI.

Focus group discussion (FGD) as a technique to collect data was chosen collectively. Throughout the FGDs, the researcher employed interview skills by probing, paraphrasing and asking questions to clarify issues.^13^ Focus group discussions was carried out in Setswana in the presence of a moderator. The researcher introduced the moderator to the caregivers at the beginning of the data collection phase. The moderator allowed caregivers to contribute meaningfully and authentically to the study. The role of the moderator in this study was to help with facilitation of data collection and ensuring the active involvement, ensuring inclusivity of caregivers. The moderator was an independent researcher from another university. The issues of confidentiality and consent forms were discussed. The researcher used an audio recorder and the caregivers were notified and consented. The discussions lasted for about 45 min–60 min. Data saturation was reached in focus group number four.

### Data analysis

Data were analysed using thematic data analysis methods. Thematic analysis is a qualitative data analysis method that involves searching a data set for repeated patterns and analysing and reporting on them.^14^ The researcher and independent co-coder used thematic analysis to analyse data. In familiarising ourselves with the data, we read and reread the transcripts. The thematic analysis determined the barriers to incorporating Ubuntu principles in IMCI. The first step of the analysis was to group related ideas by identifying and marking areas with the same units of meaning. These units of meaning were then assigned codes, the second step of thematic analysis.^15^ The data were arranged methodically and comprehensively before the researcher and co-coder began coding transcripts independently. Similar and related codes were grouped into themes based on textual phrases. Themes were checked, compared and refined to ensure they accurately represented the data with the co-coder, and the process was applied to all transcripts. The quotations from the interviews were used as citations in the findings section. The process was repeated to ensure rigour and meaning retention. The researcher and co-coder then read the information connected to each theme and determined if the information supported the theme. In the last step, the researcher and the co-coder discussed if they had the same themes and sub-themes. The co-coder was also assigned to the data analysis process to ensure that member checking or peer debriefing was conducted to ensure the findings were credible and accurately represented the data.

### Trustworthiness

In this study, the researchers adhered to the trustworthiness criteria through credibility, dependability, confirmability, transferability and authenticity to ensure quality.^14^ The trusting relationship was achieved by building a rapport with the caregivers and spending more time in the field to gain their viewpoints and ensure credibility. The study process was reported in detail to caregivers, including all research designs and how they have been implemented to ensure dependability. Confirmability was achieved by making raw data available to other researchers for member checking. An audit trail was created to provide transparency about how data were analysed and conclusions were reached, allowing others to monitor and confirm the process.^16^ Triangulation was archived when the co-authors read the transcripts and analysed data. To ensure reflexivity, the researcher’s identity, biases, assumptions and interactions with the research context shaped the study and were critically examined. A moderator was used throughout data collection to avoid bias. Data gathering methods, such as FGDs and field notes, were used to verify information.^16^ A thick description was maintained by fully indicating the research setting, techniques and co-researchers. The researcher addressed transferability by ensuring and explaining that the study results cannot be generalised to other settings and/or contexts. Authenticity was maintained and ensured by collaboration with the co-researchers, who had comprehensive and detailed experiences. It was also ensured by using direct narratives from the co-researchers.

### Ethical considerations

Ethical clearance to conduct the study was obtained from the University of Pretoria, Faculty of Health Sciences Research Ethics Committee (REC) (No. 617/2022). Written permission was obtained from the North West Department of Health (NWDoH). Permission was also obtained from the operational managers of CHC clinics and clinics of the Mafikeng sub-district in the North West province. The caregivers in the study voluntarily agreed to participate and were given an informed consent form to sign. The purpose of the study was fully explained to the caregivers. The informed consent form explained that their participation was voluntary and that they could withdraw from the study without being punished or discriminated against. For anonymity, privacy and confidentiality, caregivers were told that no names would be used, whereby only alphabets were used. The researcher gave caregivers cards with alphabets representing them to avoid using names, thus adhering to the principle of anonymity. The FGD was held in a quiet boardroom within the building, and a ‘do not disturb’ sign was placed on the door during the procedure. The use of tape recorder was explained to the caregivers.

## Results

The study focussed on the perceptions of 36 caregivers who were divided into focus groups across five healthcare facilities. The FGDs were based on the caregivers’ views on incorporating Ubuntu principles into IMCI. The results of this study are presented under themes and sub-themes (see Table 1), with quotations of the participants to support the findings of this study.

**TABLE 1:** Themes and subthemes.

Theme	Sub-themes
1 Negative attitude of professional nurses	1.1 Lack of identification of staff 1.2 Poor collaboration with caregivers 1.3 A lack of respect
2 Communication barrier	2.1 A lack of information sharing 2.2 A lack of understanding by professional nurses
3 Facilitators enhancing the incorporating of Ubuntu into IMCI	3.1 Caregivers’ health education on their health status 3.2 Proper task allocation of professional nurses in the healthcare facilities 3.3 Unannounced clinic visits by the Department of Health officials

IMCI, integrated management of childhood illness.

### Theme 1: Negative attitude of professional nurses

The findings of this study illustrated that professional nurses’ negative attitude towards caregivers is one of the barriers hampering the incorporation of Ubuntu into IMCI. This theme had three sub-themes: lack of identification of staff, poor collaboration with the caregivers and lack of respect. The sub-themes that emerged from the negative attitude of professional nurses are described next, supported by quotations from the caregivers.

#### Sub-theme 1.1 Lack of identification of staff

One of the aspects that the caregivers noticed as barriers hampering the inclusion of Ubuntu is the lack of privacy while receiving care. The caregivers highlighted that while patients are receiving care, other healthcare providers would be present in the consultation room, and it is unclear what their responsibilities are. This lack of privacy results in concerns for caregivers as they feel uncomfortable and, as a result, impedes the quality of treatment they receive. The following direct quotations confirm the finding:

‘There’s no privacy, and you are not given the feeling of comfort. Sometimes, other mothers are in the room, and you cannot answer some of the questions and end up withholding.’ (Caregiver 8, female, 36, student FGD2)

‘And there are always many nurses in the room, and we do not know their role …’ (Caregiver 6, female, 32, unemployed FGD2)

#### Sub-theme 1.2 Poor collaboration with caregivers

The caregivers expressed frustration and indicated that professional nurses would attend, examine the baby and not include them in the examination. The nurses would keep quiet and not update the mother on what they found on the child or what was wrong with the child. They would continue as if the mother or caregiver was not present. They indicated that this is one of the barriers hampering the incorporation of Ubuntu into IMCI. They elaborated:

‘… and a nurse would just take your child a mo tlhatlhobe (Examines the baby) and won’t even tell you as a mother what s/he saw or found on your child and how to further care for the baby when at home.’ (Caregiver 2, female, 27, unemployed FGD2)

‘Nurses don’t give you a chance as a mother to ask questions about your child’s health when they are done examining the child. The way I see it, they think we are questioning them.’ (Caregiver 7, female, 34, unemployed FGD3)

#### Sub-theme 1.3 A Lack of respect

The findings of this study illustrated that caregivers experience disrespectful communication from professional nurses when they bring their children for IMCI to healthcare facilities. Professional nurses mainly shout and blame them when they make mistakes with their children. The caregiver expressed the following:

‘They will shout at you, looking at your child/baby with disrespect or a belittled look. You end up not talking about your problems. When you ask for that help, they shout at you, and when you greet them, they don’t answer.’ (Caregiver 5, female, 29, unemployed FGD1)

‘On the other hand, the nurses haven’t been the same since I’ve been at this clinic. I remember one instance where I showed up on time, then got delayed as I waited in the queue and received assistance or service around 13:00. They were shouting at me, refusing to acknowledge their mistake. We argued.’ (Caregiver 1, female, 26, unemployed FGD4)

‘They shouted at me the other time when I brought my baby to the clinic, and the baby made a poop while on the scale. The professional nurse blamed me as if I said my son must do that. They must respect us. They won’t greet you or introduce themselves.’ (Caregiver 5, female, 40, unemployed FGD4)

The caregivers indicated that professional nurses are uninterested and cannot comprehend the circumstances or reasons behind caregivers’ actions. Healthcare providers are quick to judge, and some event resort to yelling at them. In most cases, they ignore caregivers and some do not even respond to greetings.

### Theme 2: Communication Barrier

The second theme in incorporating Ubuntu into IMCI was the communication barrier. The following two sub-theme emerged from this theme: a lack of information sharing and a lack of patience and understanding of caregivers by professional nurses.

#### Sub-theme 2.1 A lack of information sharing

The caregivers highlighted that they hardly get enough information concerning their children’s health. Nurses do not educate caregivers on issues concerning their children, and because of a lack of communication, they are usually afraid to ask when they have a concern:

‘Sometimes we skip the dates for immunisations because the importance is not well explained, and I wish nurses would share updates on my baby’s condition without me having to ask.’ (Caregiver 7, female, 29, cashier FGD4)

Another caregiver added:

‘I often feel left in the dark about my child’s treatment options. Clear information from nurses would help me feel in control as a mother.’ (Caregiver 7, female, 30, unemployed FGD2)

#### Sub-theme 2.2 A lack of understanding by professional nurses

The caregivers further highlighted that professional nurses lack understanding when caring for children who are under 5 years old, and this practice hinders the incorporation of Ubuntu in IMCI. In addition to affecting them and their child’s care, carers emphasised that social factors may cause them to miss vaccination or follow-up appointments. Therefore, professional nurses should be patient and understanding when explaining their circumstances. The participants stated the following in this regard:

‘Nurses, when it is lunchtime, they don’t consider that we came very early, and some of us live far from the clinic, and we walk. All of them take a break at the same time without considering us.’ (Caregiver 7, female, 30, unemplyed FGD3)

‘What I find frustrating is that they don’t get the difficulties we face as mothers – financial and social challenges that affect our clinic attendance or return visits. For example, if the grandma is taking care of the child while I am out looking for a job, she might be unable to take the child to the clinic because of their age. They should be aware of these difficulties when we clarify that sometimes there are valid reasons.’ (Caregiver 8, female, 33, unemployed FGD1)

The above-stated quotations illustrate that the negative attitudes displayed by professional nurses impact the services provided to caregivers and their children and hamper the incorporation of Ubuntu principles in IMCI.

### Theme 3: Facilitators enhancing the incorporation of Ubuntu into integrated management of childhood illness

The facilitators improving the incorporation of Ubuntu into IMCI have been identified as a theme. The following sub-themes emerged to support the main theme: caregiver’s health education on their health status, proper task allocation of professional nurses in the healthcare facilities and unannounced clinic visits by the Department of Health officials.

#### Sub-theme 3.1 Caregiver’s health education on their health status

Health education by professional nurses to caregivers was emphasised as one aspect that would enhance Ubuntu in IMCI. The caregivers expressed the need for health information on their health status and how they are expected to care for their children at home. There is a need for information concerning the health card. They were concerned, as they did not know the importance of this card. What they knew was that it was for immunisations. The following statements confirm this:

‘[*W*]e will appreciate at least if one nurse can educate us on the health issues of children under five while outside and waiting in the queue. It will show they care, and we can benefit…mmh.’ (Caregiver 5, female, 29, unemployed FGD1)

‘In addition, health talks will benefit us, and they should allow us to ask questions when we don’t understand issues concerning our children’s health because this will help us see change and seek help on time.’ (Caregiver 3, female, 26, temporary employed FGD1)

#### Sub-theme 3.2 Proper task allocation of professional nurses in the healthcare facilities

Proper daily task allocation to different programmes in the facility was referred to as one of the enablers of the incorporation of Ubuntu in IMCI. Caregivers mentioned that professional nurses often appear unhappy with the task allocation. To attend to sick children, they get irritated by minor things such as a crying baby, the nurse will then shout at the mother and say the baby is making noise. In other instances, the professional nurse will not be gentle towards their babies during vaccination. This is evident from the following direct quotation:

‘Every nurse must be placed or allocated to a task or section within the clinic according to their ability and passion because they will do their best and work harmoniously with patients. I feel a nurse who is not compassionate about children should not be placed at the child clinic as they become impatient with our children and complain that our kids are crying a lot.’ (Caregiver 4, female, 38, unemployed FGD1)

‘[*A*] nurse will attend to our children passionately because when someone does something that bores her/him, she gets irritated. You can even see how they handle our babies when we bring them for immunisation.’ (Caregiver 7, FGD 1)

‘Let nurses do what they enjoy and love so that they can enjoy working with people. Nurses will [*provide your child with undivided attention when assigned to their preferred activities*]’. (Caregiver 2, female, 24, unemployed FGD5)

#### Sub-theme 3.3 Unannounced clinic visits by the Department of Health officials

Caregivers reflected that they have never seen government officials visiting the facilities to ensure quality care and address patients’ issues with the clinic staff. Their visit must be unannounced so that it is not biased and will truly reflect what is happening. The caregivers believed that government officials’ frequent visits would enable professional nurses to provide quality care to children under 5 years old. They further indicated that this will ensure the incorporation of Ubuntu principles into the care. The following quotations are the reflection of what was said by the caregivers:

‘I sometimes wish people from the Department of Health could come and see what is happening in this clinic. I see a complaint box, but we don’t know if the complaints are addressed. Even if you can complain verbally, it is the same as the issues are not addressed. If officials can check if it is happening or if things are running smoothly… I believe these visits will make nurses practice Ubuntu, such as respecting and giving us information.’ (Caregiver 5, female, 40, unemployed FGD6)

‘And when they come, they must not announce their visit. It must be a surprise because if they get a call, they are coming, and everything will be done to provide care promoting human dignity. We are afraid to complain verbally because they end up victimising you, so if we can have an observer outside the clinic to see how we are treated.’ (Caregiver 1, female, 29, unemployed FGD2)

The aforementioned quotations illustrated that the enablers enhancing the incorporation of Ubuntu into IMCI are necessary to provide quality care to children under 5 years old. The enablers will facilitate caregivers’ engagement in decision making and collaboration on their children’s health. By employing these facilitators, the healthcare industry would improve its care offerings and successfully improve patient outcomes and employee satisfaction.

## Discussion

The study aimed to explore and describe the barriers and facilitators of incorporating Ubuntu principles in managing childhood illnesses.^17^ The findings of this study illustrated that the negative attitude of professional nurses towards caregivers hinders the incorporation of Ubuntu principles in IMCI. The negative attitudes alluded to by caregivers include professional nurses not explaining the health status of their children to them, the absence of privacy during consultations, nurses’ lack of care and disrespectful conduct. The negative attitudes also include a lack of engagement and cooperation between caregivers and professional nurses. Patients frequently base their expectations of healthcare on Ubuntu principles.^17^ However, nurses’ unfavourable attitudes towards caring are not motivated by Ubuntu principles.^18^ Furthermore, adverse reports about nursing treatment that lack Ubuntu creates a void in the caring relationship between nurses and patients.^11^ In agreement, the data demonstrated that caregivers believe nurses’ negative attitudes should be addressed to facilitate the incorporation of Ubuntu principles into IMCI.

According to the caregivers, healthcare professionals treat them disrespectfully, are indifferent and impatient, and do not engage them. Since Ubuntu ethics demands free and informed consent regardless, respecting an individual is essential to maintaining their dignity.^9^ The lack of respect hinders the quality of care. Literature further revealed that the lack of respect also increases uncertainty.^19^ The lack of respect is the absence of professional behaviour and Ubuntu. Literature indicated that nurses have difficulty in respecting complex patients who are rude, aggressive and demanding.^20^ In addition, in solidarity with the latter, literature argues that by putting egos aside, the community may show confidence in the process and Ubuntu principles would thrive.^21^ Literature highlights that the healthcare personnel know the right to confidentiality and privacy.^22^ Nurses in Limpopo expressed challenges in implementing the IMCI in healthcare facilities and indicated that the infrastructure seems to interfere with the provision of privacy.^23^ Researchers indicate that nurses need to adhere to their professional code of ethics and ethical guidelines to ensure the well-being and safety of their patients.^24^ One implication for nursing practice is the importance of maintaining patient confidentiality. Furthermore, nurses must be prepared to handle ethical challenges in their practice, including conflicts of interest, cultural differences and moral dilemmas. Nurses should have the knowledge and skills to navigate ethical challenges and seek guidance from committees or supervisors as needed.

In order to improve the care experience in healthcare facilities, ethical issues are critical in shaping the quality of patient care, the well-being of healthcare workers and the overall effectiveness of the healthcare system. Nurses experiencing burnout may exhibit anger, fail to complete assigned tasks and appear incompetent in patient care. Patients may perceive these actions as contradicting the values of caring and compassion in the Ubuntu philosophy.^17^

Ngondo and Klyueva specified that individuals may reject Ubuntu if they do not feel they will not benefit from a collective action.^25^ Thus, practising Ubuntu values and principles influence the need for collaboration and providing respectful care. Interconnectedness and information sharing are the core values of Ubuntu, and they are essential to empower caregivers. It was further argued that communal relationships involve constructive solidarity among diverse individuals and assist in the quality of care for all.^9^ The Ubuntu philosophy reiterates the collaboration of human beings as one of the values. Collaboration and participatory decision making of professional nurses and caregivers would foster the provision of care at home, yielding optimal results.^26^

The study also found that incorporating Ubuntu principles into IMCI is hampered by communication gaps. Caregivers reported that professional nurses do not share enough information with them or adequately educate them on health related issues. They also emphasised that professional nurses do not comprehend the financial and social circumstances that caregivers face.

In addition to a heavy workload, professional difficulties and conflicts put pressure on nurses to interact and communicate poorly with patients.^10^ Without knowing one another, people would converse in many public settings in a polite and friendly manner.^27^ Their shared humanity appears to be the only thing that unites them; in other words, their unrestricted relations happen because they co-belong.^27^

The findings of the study further highlight facilitators that can enhance the incorporation of Ubuntu principles in IMCI. The caregivers mentioned that health education on their status, treatment plans, medication and any relevant information concerning their health will benefit them and their children. Proper daily task allocation for nurses incorporating Ubuntu will improve professional nurses’ attitudes.

Ngubane and Makua argue that based on the values of Ubuntu philosophy, respect, caring, sharing and compassion, Ubuntu does not necessarily focus on sharing what you have; instead, respecting fellow human beings in the community brings harmony. Respect brings about cooperation among people.^5^ Collaboration of different stakeholders promotes a sense of belonging.^11^ Furthermore, information sharing reflects compassion and improves collaborations among people. Collaboration is essential when providing care.^28^ Incorporating Ubuntu ideals into IMCI requires nurses to practice privacy and involve caregivers in caring for their children. Furthermore, Ubuntu’s caring values must be included for everyone’s success.^11^ People who respect each other and come together, solve problems. Therefore, incorporating Ubuntu into IMCI involves respectful and loving nurses, sharing information and providing care with dignity.

The findings suggest a need to include enablers to facilitate the incorporation of Ubuntu principles in IMCI, as this would necessitate professional nurses reflecting on the history of caring. Nurses providing Ubuntu care are relational, providing a suitable environment to interact with their patients and allowing them to converse within safe boundaries.^29^ Therefore, when Ubuntu principles are in place, there will be understanding and respect among professional nurses and caregivers of children under 5 years old.

### Strengths and limitations of the study

This study provided a broader understanding and insight into barriers and facilitators incorporating Ubuntu principles in IMCI. The findings support the incorporation of Ubuntu principles into the healthcare system. The limitation of the study is that findings cannot be generalised to a broader population as it was conducted in specific clinics in the Mafikeng sub-district of the North West province. Only one sub-district in the province was used to gather data and contextualise the study. Although the conclusions of the study cannot be generalised, they can be used in other contexts to enhance the implementation of IMCI.

### Recommendations

The recommendations were made in the context of nursing practice, nursing education and nursing research.

It is recommended that the clinical practice environments adopt the incorporation of Ubuntu principles in the management of childhood illness. All healthcare workers should be required to receive ongoing ethics, compassion, and cultural competency training and certification from government health bodies and professional nursing associations. Ethical concerns regarding nursing care, compassion, Ubuntu and research on practitioners’ perspectives must be addressed to ensure that both care providers and recipients experience an environment of mutual respect, dignity and fairness.

Nursing schools should revise and expand their curricula to include more comprehensive training in ethics, compassion and Ubuntu. This includes not only theoretical knowledge but also practical case studies, role-playing and reflective practices that prioritise empathy, shared responsibility and patient-centred care including IMCI.

The study recommends that the success stories of incorporation of Ubuntu principles in the management of childhood illness should be documented. As a result, it is recommended that more research could investigate the impact of incorporating Ubuntu principles into nursing care. Researchers can look into how Ubuntu’s emphasis on interconnectedness, empathy and collective well-being affects healthcare professionals’ attitudes and behaviours, particularly in terms of patient-centred care.

## Conclusion

The study showed barriers to incorporating Ubuntu in IMCI. It also demonstrated that the incorporation of Ubuntu in IMCI is essential. The findings of the study support the need for healthcare providers to prioritise the Ubuntu principles for the better management of childhood illness for children under 5 years old. The study further outlined the facilitators for incorporating Ubuntu principles into IMCI. The study also supports the creation of favourable environments that can make institutional changes to support holistic, community centred care for children under 5 years old based on Ubuntu principles. Facilitators such as emphasis on community care, holistic health and empathy can help overcome some of these barriers, resulting in improved health outcomes for children under 5 years old, particularly when traditional and healthcare practices are aligned. Efforts to integrate Ubuntu principles would necessitate education, collaboration and changes across the system to bridge the gap between various healthcare philosophies and promote children’s well-being in a more inclusive and holistic way. Nursing personnel must be educated and trained more on important topics such as ethics, compassion and Ubuntu in order to create a more holistic and patient-centred healthcare environment. Furthermore, understanding the perspectives of healthcare practitioners through research is critical for creating an environment in which Ubuntu principles can be more effectively integrated into care practices.
